# Xuezhikang, Extract of Red Yeast Rice, Improved Abnormal Hemorheology, Suppressed Caveolin-1 and Increased eNOS Expression in Atherosclerotic Rats

**DOI:** 10.1371/journal.pone.0062731

**Published:** 2013-05-10

**Authors:** Xin-Yuan Zhu, Pei Li, Ya-Bing Yang, Mei-Lin Liu

**Affiliations:** Department of Geriatrics, Peking University First Hospital, Beijing, China; Max Delbrueck Center for Molecular Medicine, Germany

## Abstract

**Background:**

Xuezhikang is the extract of red yeast rice, which has been widely used for the management of atherosclerotic disease, but the molecular basis of its antiatherosclerotic effects has not yet been fully identified. Here we investigated the changes of eNOS in vascular endothelia and RBCs, eNOS regulatory factor Caveolin-1 in endothelia, and hemorheological parameters in atherosclerotic rats to explore the protective effects of Xuezhikang.

**Methodology/Principal Findings:**

Wistar rats were divided into 4 groups (n = 12/group) group C, controls; group M, high-cholesterol diet (HCD) induced atherosclerotic models; group X, HCD+Xuezhikang; and group L, HCD +Lovastatin. In group X, Xuezhikang inhibited oxidative stress, down-regulated caveolin-1 in aorta wall (*P*<0.05), up-regulated eNOS expression in vascular endothelia and erythrocytes (*P*<0.05), increased NOx (nitrite and nitrate) in plasma and cGMP in erythrocyte plasma and aorta wall (*P*<0.05), increased erythrocyte deformation index (EDI), and decreased whole blood viscosity and plasma viscosity (*P*<0.05), with the improvement of arterial pathology.

**Conclusions/Significance:**

Xuezhikang up-regulated eNOS expression in vascular endothelia and RBCs, increased plasma NOx and improved abnormal hemorheology in high cholesterol diet induced atherosclerotic rats. The elevated eNOS/NO and improved hemorheology may be beneficial to atherosclerotic disease.

## Introduction

Xuezhikang, the extract of red yeast rice, has been widely used as a Chinese traditional medicine for the therapy of patients with cardiovascular diseases. It contains natural Lovastatin and its homologues, as well as unsaturated fatty acids, flavonoids, plant sterols and other biologically active substances [1]. Previous clinical studies showed that Xuezhikang could exert anti-inflammatory actions [2] and improve endothelial function [3], but the exact molecular mechanisms of anti-atherosclerosis have not been fully elucidated.

Nitric oxide (NO) is a key regulator of endothelial function and vascular homeostasis [4], [5]. Endothelial NO possesses multiple antiatherosclerotic properties, including inhibition of leukocyte adhesion and prevention of smooth muscle proliferation [6]. Previous study has shown that eNOS deficiency accelerates the development of atherosclerosis in apolipoprotein E-knockout mice [7]. Caveolae, the flask-shaped invaginations of the plasma membrane, are the predominant locations of eNOS [8] in endothelial cells. In caveolae of endothelial membrane, eNOS is co-localized with caveolin-1 (Cav-1), the major coat protein of caveolae, and the interaction of eNOS and Cav-1 maintains the eNOS in its inactive conformation [8]–[10]. In Cav-1^−/−^ ApoE^−/−^ mice, atherosclerotic plaque area is markedly reduced despite the presence of hypercholesterolemia [11]. Therefore, eNOS, Cav-1 and their interactions may be a novel target site for therapy of atherosclerosis.

In addition to controlling vascular tone, NO is also a potent regulator of hemorheology mainly due to the improvement of erythrocyte rheological properties [12], [13]. There are evidences that hemorheological abnormalities play an important role in the pathogenesis and development of atherosclerosis [14]–[18]. Therefore, improving hemorheology may be one of the mechanisms explaining the antiatherosclerotic effects of NO. Although it is generally considered that RBCs act as “NO sink” via the NO-scavenger oxyhemoglobin, RBCs are also proved to express functional eNOS and produce NO by themselves via eNOS under normoxic conditions [19]–[22]. RBC eNOS may be involved in regulating RBC lifespan [23], membrane fluidity of erythrocytes [24], erythrocyte deformability [12], [13], platelet aggregation [25], and blood flow [26] through the production of NO, thus affecting hemorheology. Besides, in patients with coronary artery disease, RBC eNOS expression and activity are both lower than in age-matched healthy individuals and correlate with the degree of endothelial dysfunction [20]. Therefore, RBC eNOS expression may be another target for treatment of atherosclerotic diseases.

In order to explore the molecular mechanisms of anti-atherosclerotic effects of Xuezhikang, we investigated the changes of eNOS in vascular endothelia and RBCs, NOS regulatory factor Cav-1 in endothelia, and hemorheological parameters after Xuezhikang treatment in high cholesterol diet induced atherosclerotic rats.

## Materials and Methods

### Preparation of the Drug Xuezhikang

Xuezhikang powder was provided by WBL Peking University Biotech Co., Beijing, China. Xuezhikang is the mixture of 13 natural monacolins, such as monacolin K, L, J, M and X. The structure of monacolin K in its lactone form is identical to lovastatin, and lovastatin always be used as the quality standard for Xuezhikang. Xuezhikang contains 0.8% Lovastatin, 8% unsaturated fatty acids (primarily linoleic acid, oleic acid, palmitic acid, stearic acid, etc), as well as essential amino acids, ergosterol and some other components.

### Animal Treatment

Animal study was performed in strict accordance with the Care and Use of Laboratory Animals recommended by National Institute of Health, and was approved by the Animal Research Committee of Peking University Health Sciences. Animal surgery was carried out under sodium pentobarbital anesthesia.

Wistar rats of 200±20 g body weight from the Animal Center of Academy of Military Medical Sciences were randomly assigned into four groups (n = 12/group): (1) controls (group C), (2) fed with high cholesterol diet (HCD), atherosclerotic models (group M), (3) HCD+Xuezhikang (group X), (4) HCD+Lovastatin (group L). Rats in groups M, L and X were intraperitoneally injected with a single dose of vitamin D_3_ (600,000 U/kg) and fed with the high-cholesterol diet (containing 3% cholesterol, 0.5% cholate, 5% refined sugar, 10% lard and 0.2% propylthiouracil) to induce atherosclerosis. Lovastatin and Xuezhikang were administered by gastric tube with the dose of 2.5 mg·kg^−1^·d^−1^ and 300 mg·kg^−1^·d^−1^, respectively, based on the doses used in previous studies [27] and the body surface area normalization method. Rats in group C were fed with normal rat chow with tube feeding of same volume of saline daily. The experiment lasted for 12 weeks.

### Blood and Tissue Samples

At the end of the experiment, rats were anesthetized by sodium pentobarbital (1.25 g/kg) after overnight fasting. Blood was collected by abdominal aorta puncture and anticoagulated with heparin for hemorheological examinations.

Anticoagulated blood was centrifuged 3,000 rpm for 10 min at 4°C to separate plasma and erythrocytes. Plasma samples were used for the measurements of Triglyceride (TG), low density lipoprotein cholesterol (LDL-C), NOx (total nitrite and nitrate), MDA, SOD and T-AOC. Packed erythrocytes were washed twice with heparin-phosphate-buffered saline (PBS), and added to five volumes of hypotonic buffer (10 mmol/L Tris-HCl, pH 7.4) to make hemolysis. After centrifugation, the supernatant was collected as erythrocyte cytoplasm for cGMP determination. The pellet was washed and centrifuged three times to obtain erythrocyte membrane sample. The erythrocyte membrane sample was lysed in sample buffer (20 mmol/L Tris pH 7.4, 2.5 mmol/L EDTA, 1% Triton X-100, 1% deoxycholic acid, 0.1% sodium dodecyl sulfate, 100 mmol/L NaCl, 10 mmol/L NaF, and protease inhibitor cocktail) for eNOS determination by western blotting.

Aorta between the aortic valve cusps and bifurcation at iliac arteries was isolated and taken off the gross adventitial tissue. The ascending aorta was quickly fixed in 4% formaldehyde for histological studies. Aortic arch was homogenized in RIPA buffer and centrifuged at 12,000 rpm for 20 minutes at 4°C. Protein lysates of aortic arch were used for the measurements of eNOS, p-eNOS and caveolin-1 by western blotting, the measurement of cGMP by ELISA, and immunoprecipitation. Protein concentration in aortic arch lysate and erythrocyte membrane sample was assayed by a BCA protein quantification kit (KeyGen Biotechnology, China).

### Hemorheological Parameters

Rat blood samples were measured for erythrocyte deformation index (EDI), whole blood viscosity (WBV), plasma viscosity (PV), and hematocrit (Hct). EDI was determined at various fluid shear stresses by using an ektacytometer (Model LBY-BX2, Precil Co., China). Erythrocytes were re-suspended in 15% polyvinylpyrrolidone (PVP, MW 30 kDa) buffer (61 mM NaCl, 0.8 mM Na_2_HPO_4_, 0.2 mM KH_2_PO_4_, pH 7.4, 290 mOsm/kg, viscosity 15 mPa·s) and adjusted to 2×10^7^ cells/ml to measure EDI. The EDI was calculated for shear rates from 50 to 1000 s^−1^. Whole blood viscosity was measured by using a cone-plate viscometer (Model LBY-N6A, Precil Co., China). Plasma viscosity was measured with a capillary viscometer (Model LBY-NM2, Precil Co., China). Hematocrit was measured in a capillary tube after centrifugation (15,000 rpm for 3 min) by a microhematocrit centrifuge. All measurements were carried out at 37°C according to the International Guidelines for the measurements of hemorheologic parameters.

### Plasma NOx (Total Nitrite and Nitrate), MDA, SOD and T-AOC

NO is chemically active and rapidly converted into nitrate (NO3^−^) and nitrite (NO2^−^) in vivo. NO2^−^ is further converted into NO3^−^. In this study, specific nitrate reductase method and Griess reaction were performed to detect NO metabolites (nitrite and nitrate).

Malondialdehyde (MDA) was analyzed by TBA assay. SOD was measured by degree of inhibition on nitroblue tetrazolium (NBT) produced by superoxide radicals generated from the xanthine/xanthine oxidase system. Total antioxidant capacity (T-AOC) in plasma was measured based on the level of Fe^3+^ reduced to Fe^2+^. We followed the instruction of the kit (Nanjing Jiancheng Biology Engineering, China), and measured using an ultraviolet/visible scanning spectrophotometer.

### Western Blotting for eNOS, Phospho-eNOS, and Caveolin-1

Equal amount of proteins were separated by SDS-polyacrylamide gel (SDS-PAGE) electrophoresis at 4°C, and the proteins were transferred onto a nitrocellulose membrane. After blocking nonspecific sites, the membrane was incubated with anti-eNOS antibody (1∶1000 sc-376751, Santa Cruz Biotech), anti-eNOS p-ser 1177 antibody (1∶500, Santa Cruz Biotech), anti-tubulin antibody (1∶1500, Santa Cruz Biotech), anti-caveolin-1 antibody (1∶1000, Santa Cruz Biotech), or anti-β-actin antibody (1∶1500, Santa Cruz Biotech) overnight. Blotted antibodies were detected by a horseradish peroxidase-labeled second antibody (1∶8000, Santa Cruz Biotech) and enhanced chemiluminescent technique. Density of the bands was measured by an image analyzer.

### Co-immunoprecipitation of eNOS with Caveolin-1 in Aorta Wall

Protein lysate of aorta wall was pre-cleared by incubation with 50 µl of 50% (V/V) protein A sefinose (Sangon Biotech Co., China) for 30 min to pre-clean the lysate. 5 µl of anti-caveolin-1 antibody (Santa Cruz Biotech, CA, USA) and 50 µl of 50% (V/V) protein A sefinose were added to the pre-cleaned supernatant and the mixture was incubate at 4°C for 5 hours on a shaker. The resin was collected by centrifugation at 2,500 rpm at 4°C for 1 min, washed with 1 ml PBS for 3 times, and re-suspended in 80 µl loading buffer. After heated at 100°C for 5 min and centrifugation, the samples were used for western blotting to detect eNOS by the anti-eNOS antibody.

### Immunohistochemical Detection of eNOS and Caveolin-1 in Aorta

Longitudinal sections of ascending aorta were used for the examinations. The slides were heated in a microwave oven for 15 min in 10 mM sodium citrate buffer pH 6.0 and then incubated in the buffer at room temperature for 20 min to retrieve antigens. After washing, the slides were incubated in 0.3% H_2_O_2_ in 100% methanol for 30 min to inactivate endogenous biotin. After washing with PBS and blocking in 1.5% normal goat serum, the slides were then incubated with anti-eNOS monoclonal antibody (1∶100) or anti-caveolin-1 polyclonal antibody (1∶100) overnight at 4°C. After washing with PBS, the slides were sequentially incubated with biotinylated goat anti-mouse IgG or goat anti-rabbit IgG antibody, HRP-conjugated streptavidin and development reagent. A high power microscope with digital imaging system was used for photograph after counterstaining the slides with hematoxylin.

### ELISA for cGMP in Aorta Wall and Erythrocyte Cytoplasm

Hemoglobin in erythrocyte cytoplasm was removed by an ultrafilter (cutoff 50-kDa, Millipore) as described previously [28]. ELISA for cGMP was performed using a commercially available kit specific for rat cGMP (Cusabio Biotech Co., China) following manufacture’s instruction.

### Statistical Analysis

Results were presented as means±standard deviations. Statistical differences were evaluated by one-way analysis of variance (ANOVA) with Tukey or Dunnett post hoc analysis. A value of *P*<0.05 was considered to be statistically significant.

## Results

### Plasma Lipids

In group M, plasma LDL-C was significantly increased as compared with that in group C (*P*<0.05). In group X, plasma TG and LDL-C were significantly decreased as compared with those in group M (*P*<0.05). There were no significant differences in plasma lipid levels between groups X and L (Table 1).

**Table 1:** Plasma lipids in the four groups of rats (mean±SD).

Group (n = 12/group)	TG(mmol/L)	LDL-C(mmol/L)	HDL-C(mmol/L)
M	0.67±0.19	6.43±1.93*	1.12±0.32*
L	0.31±0.11	3.69±1.08	0.96±0.33
X	0.29±0.16**	3.32±0.29**	0.94±0.31
C	0.64±0.06	0.28±.012	0.64±0.06

* *P*<0.05 vs. group C;

** *P*<0.05 vs. group M.

M: atherosclerosis model group, rats were fed with high cholesterol diet (HCD); L: Lovastatin group, rats were fed with HCD+Lovastatin; X: Xuezhikang group, rats were fed with HCD+Xuezhikang. C: control group, rats were fed with regular diet.

### Oxidative Stress Parameters

In group M, plasma MDA increased and SOD and T-AOC decreased as compared with those of group C (*P*<0.05). In groups L and X, plasma MDA decreased and SOD and T-AOC increased as compared with those in group M (*P*<0.05). The changes of MDA, SOD and T-AOC were more significant in group X than in group L (*P*<0.05), also suggesting that Xuezhikang is more effective than lovastatin in alleviating oxidative stress in rats with hyperlipidemia and atherosclerosis (Table 2).

**Table 2:** Plasma malondialdehyde (MDA), superoxide dismutase (SOD) and total antioxidant capacity (T-AOC) in the four groups of rats (mean±SD).

Group (n = 12/group)	MDA (nmol/ml)	SOD (U/ml)	T-AOC (U/ml)
M	11.52±1.98*	62.39±12.08*	6.64±2.31*
L	9.62±0.48**	72.13±4.75**	8.05±1.03**
X	8.68±0.40** #	95.57±4.15** #	8.57±0.96** #
C	6.23±0.63	107.7±6.07	9.08±0.54

* *P*<0.05 vs. group C;

** *P*<0.05 vs. group M;

# *P*<0.05 vs. group L.

### Pathological Changes in Aorta Wall

**Figure 1:**
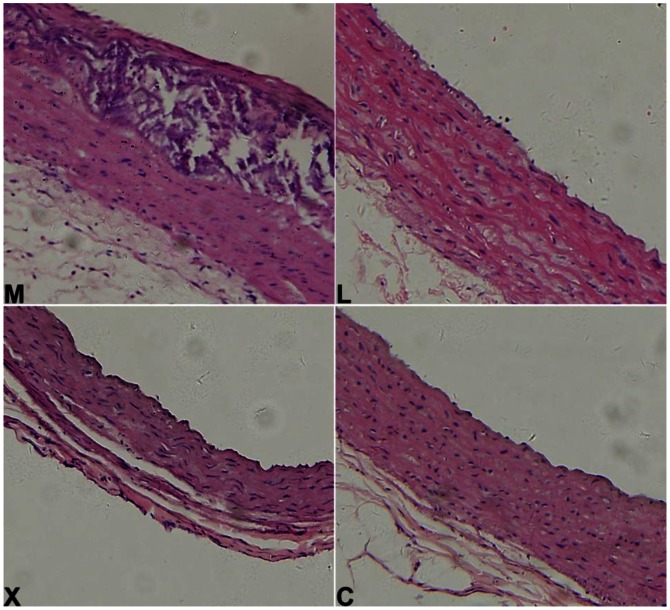
Pathological changes of ascending aorta of rats from groups M, L, X and C. (H-E staining, 20x).

Microscopic examination of the ascending artery revealed no pathological changes in group C. Rats in group M developed typical plagues with macrophage infiltration and thickened intima. In group X, the morphology of aorta was comparable to that of group C (Figure 1), indicating the remarkable effects of Xuezhikang in prevention of rats with high cholesterol diet induced atherosclerosis.

### Hemorheological Parameters

In group M, erythrocyte deformation index (EDI) decreased and whole blood viscosity (WBV) and plasma viscosity (PV) increased as compared with those in group C (*P*<0.05). In group X, EDI increased and WBV and PV decreased as compared with those in group C (*P*<0.05). The increase of EDI and decrease of WBV and PV were more significant in group X than in group L (*P*<0.05), suggesting that Xuezhikang is more effective than lovastatin in improvement of hemorheological parameters in rats with hyperlipidemia and atherosclerosis (Table 3).

**Table 3:** Hemorheological parameters in the four groups of rats (mean±SD).

	WBV (mPa·s)			EDI (max)	Pv (mPa·s)	HCT
Group (n = 12/group)	50/S^−1^	100/S^−1^	150/S^−1^			
M	5.47±0.41*	4.91±0.39*	4.58±0.33*	0.31±0.02*	1.81±0.16*	0.41±0.01
L	4.66±0.59	4.17±0.45	3.97±0.39	0.32±0.01	1.68±0.16	0.41±0.01
X	4.50±0.26** #	4.04±0.19** #	3.87±0.20** #	0.35±0.01** #	1.54±0.20** #	0.41±0.02
C	4.72±0.68	4.34±0.64	3.99±0.48	0.36±0.01	1.56±0.15	0.42±0.01

* *P*<0.05 vs. group C;

** *P*<0.05 vs. group M;

# *P*<0.05 vs. group L.

### Plasma NOx Level

**Figure 2:**
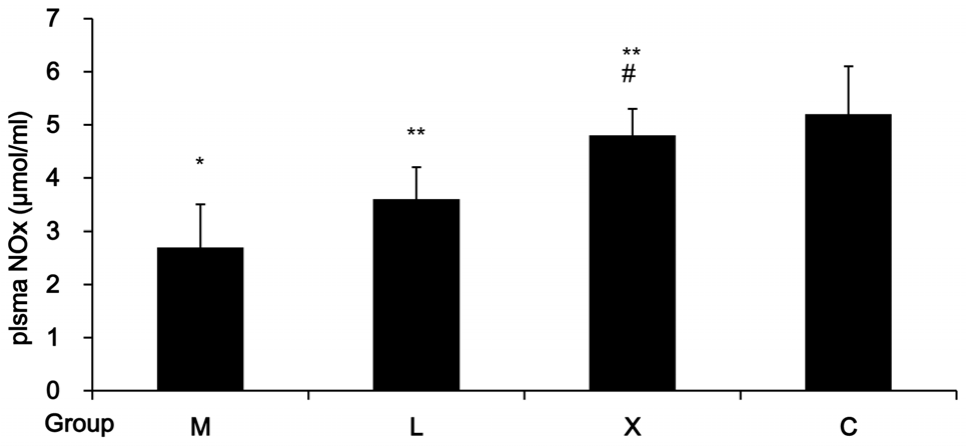
Effect of Xuezhikang on plasma NOx (nitrite and nitrate). (n = 12/group). *:*P*<0.05 vs. group C; **:*P*<0.05 vs. group M; #:*P<*0.05 vs. group L.

Plasma NO metabolites (nitrite and nitrate; NOx) were measured to estimate NO production. Plasma NOx increased significantly in groups X and L as compared with that in group M (*P*<0.05), and was higher in group X than in group L (*P*<0.05), suggesting that Xuezhikang is more effective than lovastatin in promoting NO production in rats with hyperlipidemia and atherosclerosis (Figure 2).

### cGMP Concentration in Aorta Wall and Erythrocyte Cytoplasm

**Figure 3:**
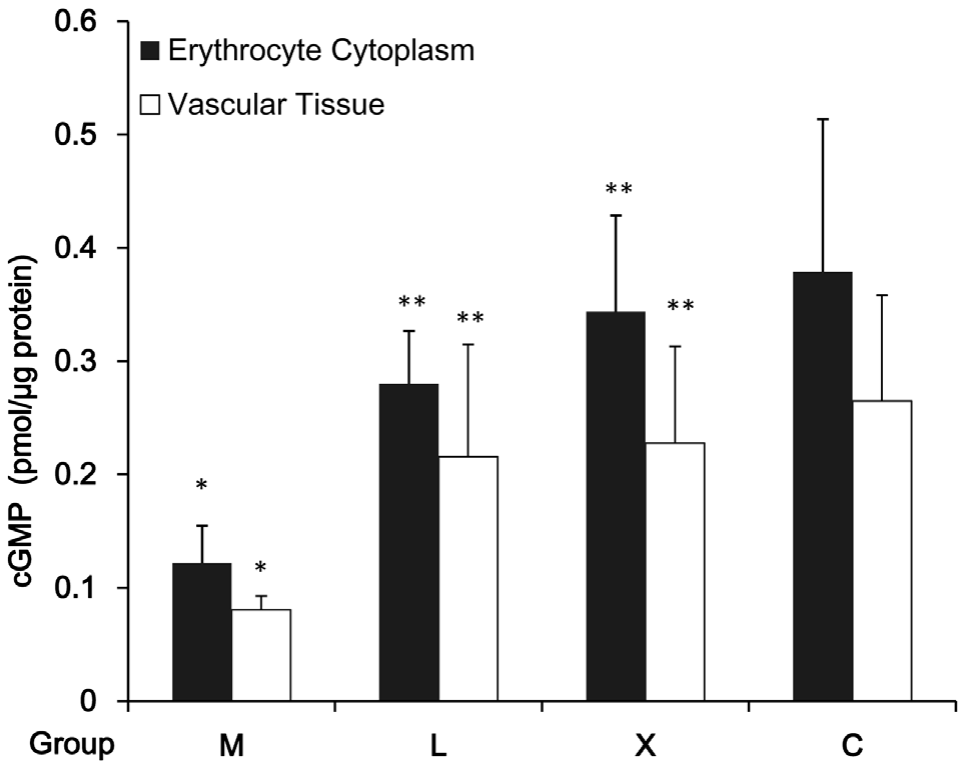
Effects of Xuezhikang on cGMP level in aorta wall and erythrocyte cytoplasm. Hemoglobin in erythrocyte cytoplasm had been removed by ultrafiltration before the assay. *:*P*<0.05 vs. group C; **:*P*<0.05 vs. group M.

In group M, cGMP level in aorta wall and erythrocyte cytoplasm decreased as compared with that in group C (*P*<0.05). In groups X and L, cGMP level in these two sites increased as compared with those in group M (*P*<0.05, Figure 3). cGMP synthesis is activated by many factors, of which the important one is NO stimulation.

### eNOS Expression on Erythrocyte Membrane

**Figure 4:**
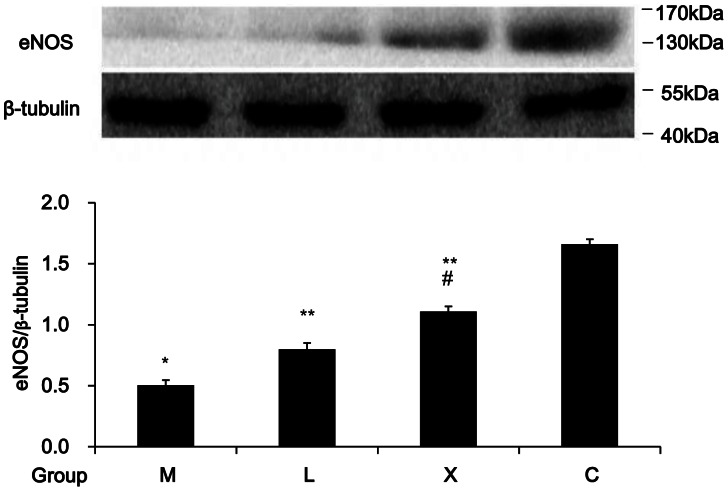
Effect of Xuezhikang on eNOS expression on erythrocyte membrane (n = 6/group). About a 134 kDa band was identified by anti-eNOS antibody. β-tubulin was used as an internal reference. *:*P*<0.05 vs. group C; **:*P*<0.05 vs. group M; #:*P*<0.05 vs. group L.

eNOS was expressed on rat erythrocyte membrane (see Figure S1). In group M, eNOS on erythrocyte membrane decreased as compared with that of the group C. eNOS on the membrane increased in groups X and L as compared with that in group M, and was higher in group X than in group L (*P*<0.05), suggesting that Xuezhikang is more effective than lovastatin in inducing eNOS expression on erythrocytes in rats with hyperlipidemia and atherosclerosis (Figure 4). However, we did not measure the eNOS activity and its changes on erythrocytes.

### eNOS Expression in Aorta Wall

**Figure 5:**
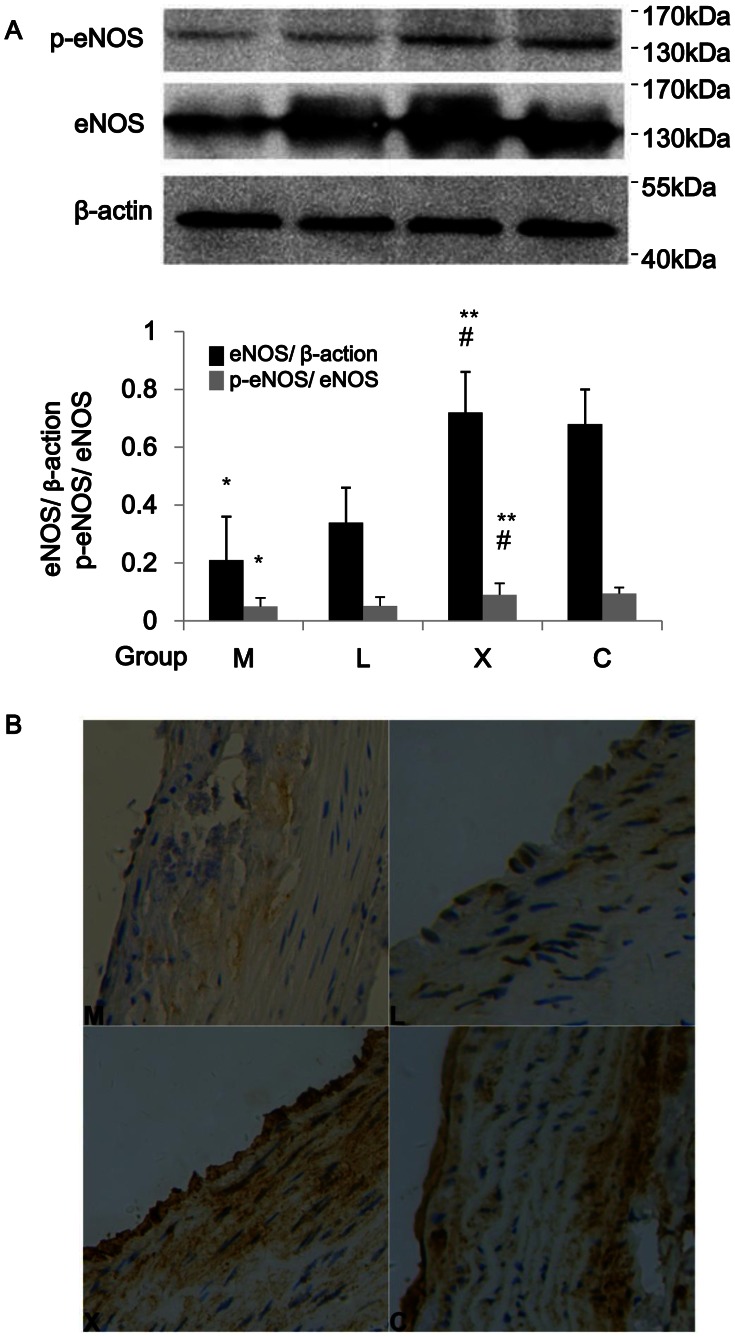
Effects of Xuezhikang on eNOS expression in aorta. (A) Protein lysate of aorta wall was used for the detection of eNOS, eNOS p-ser1177 by western blotting (n = 6/group). *:*P*<0.05 vs. group C; **:*P*<0.05 vs. group M; #:*P*<0.05 vs. group L. (B) Immunohistochemistry of aorta wall located eNOS expression in aorta endothelia (brown staining).

In group M, eNOS and p-eNOS in aorta wall decreased as compared with that of group C. In group X, eNOS and p-eNOS was significantly higher than those in group C and group L (*P*<0.05, Figure 5A), and the increase of eNOS predominantly located in aortic endothelia (Figure 5B). Additionally, changes of the ratio of p-eNOS/eNOS were similar to the changes of total e-NOS (Figure 5A). Therefore, Xuezhikang is more effective than lovastatin in inducing eNOS expression in aorta wall in rats with hyperlipidemia and atherosclerosis.

### Caveolin-1 Expression in Aorta Wall

**Figure 6:**
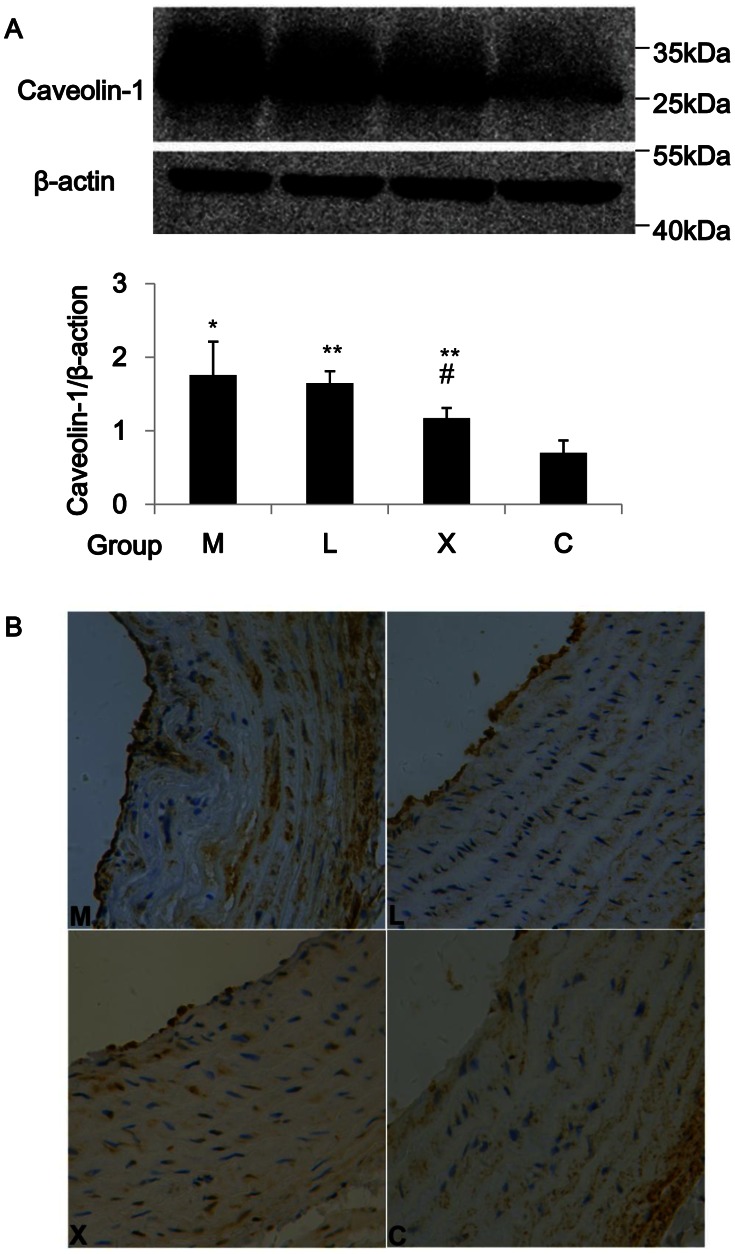
Effect of Xuezhikang on caveolin-1 expression in aorta wall. (A) Protein lysate of aorta wall was used for the detection of caveolin-1 (n = 6/group). *:*P*<0.05 vs. group C; **:*P*<0.05 vs. group M; #:*P<*0.05 vs. group L. (B) Immunohistochemistry of aorta wall located caveolin-1 predominantly in aortic endothelia (brown staining).

**Figure 7:**
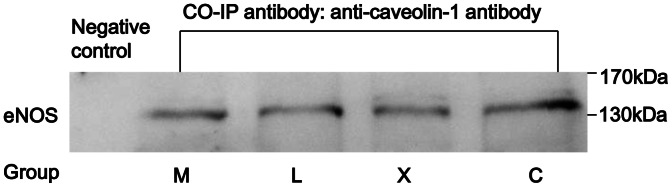
Interaction between eNOS and caveolin-1 in aorta wall. Protein lysates of aorta wall were incubated with anti-caveolin-1 antibody and protein A resin, and the immuno-precipitated proteins were blotted by anti-eNOS antibody by western blotting. Negative control sample was taken from the mixed supernatant from groups M, L, X and C, and was subjected to immunoprecipitation without the addition of anti-caveolin-1 antibody.

Caveolin-1 expression changed reversely with eNOS expression in aorta wall among the four groups, i.e., caveolin-1 increased in group M, decreased in groups L and X, and the decrease was more significant in group X than in group L (Figure 6A). Immunohistochemistry of aorta wall confirmed the caveolin-1 changes by western blotting (Figure 6B). Immunoprecipitation assay demonstrated that eNOS and caveolin-1 were closely interacted in aorta wall (Figure 7). Xuezhikang induced decrease of caveolin-1 may result in the activation of eNOS through which NO production may be increased from aortic endothelia.

## Discussion

There is no plasma cholesterylester transfer protein (CETP) in rats, and 80% of total plasma cholesterol exists in HDL particles. Therefore, rats are considered to be resistant to atherogenesis and require higher cholesterol diet to form arterial plagues [29]. A high-cholesterol diet combined with a single dose of vitamin D_3_ (600,000 U/kg) [30], [31] can be used to induce atherosclerosis in rats.

In this study, we found that Xuezhikang increased eNOS expression in vascular endothelia and erythrocytes, and decreased the expression of caveolin-1 in aorta. These changes are expected to increase NO production, which was confirmed by the increase of NOx (nitrate and nitrite) in plasma and cGMP in aorta wall. Besides, Xuezhikang treatment ameliorated the hemorheological abnormalities and oxidative stress, lowered the higher serum lipids, and improved the pathology of atherosclerosis.

Previous studies showed that the cholesterol synthesis inhibitor rosuvastatin can increase eNOS activity in endothelial cells [32]. In our study, Xuezhikang also increased the eNOS expression in aortic endothelia in association with the decrease of plasma lipids. Besides, Xuezhikang was more potent than Lovastatin in rat atherosclerosis model. We speculated that in addition to the natural lovastatin, other useful constituents such as statin homologues, unsaturated fatty acids, essential amino acids and ergosterol may also be capable of promoting eNOS/NO production. Furthermore, caveolin-1 expression in aorta increased in atherosclerotic rats and decreased in Xuezhikang treatment rats, which was reversely correlated to eNOS expression. Our findings support the hypothesis that reduction of NO in atherosclerotic model may be the consequence of enhanced expression of caveolin-1.

RBCs also contain functional eNOS [19]–[22], which is localized on the cytoplasmic side of membrane [19]. Under normoxic conditions, NO synthesis in RBCs is largely NOS-dependent [20], [33] and is comparable to that of cultured human endothelial cells [19]. However, under hypoxic conditions, NO production is probably independent of NOS activity [34], and S-nitrosohemoglobin (SNO-Hb) [35]–[37], iron-nitrosylhemoglobin [38] or nitrite may be the sources of NO to attenuate hypoxia through NO-dependent increase of blood flow [23]. Our study found that eNOS on RBC membrane and NO metabolites in plasma increased significantly after Xuezhikang treatment. The significance of the increased eNOS on erythrocytes is difficult to be evaluated due to the facts that we did not measure the eNOS activity of erythrocytes, and the increased plasma NO may originate from multiple sources such as endothelia. Besides, it is generally considered that mature red blood cells are incapable of protein synthesis, but we found the increase of eNOS in RBCs after the treatment. Presumably, erythroid progenitor cells in bone marrow express higher eNOS protein after Xuezhikang treatment, resulting in the increase of eNOS on cell membrane of circulating mature RBCs. For example, Cokic et al. showed that erythroid progenitor cells (EPCs) contained eNOS mRNA and protein, and hydroxyurea was an inducer of the NO/cGMP pathway in EPCs [39]. Bone marrow examinations may be useful to understand the source of increased eNOS on mature RBCs after Xuezhikang treatment.

Hemorheological status is determined by many factors including erythrocyte deformability, blood volume, plasma viscosity, platelet activation, etc. Hemorheological abnormalities play an important role in the pathogenesis and development of atherosclerosis [14]–[18]. In the presence of abnormal hemorheology, more leucocytes and platelets are concentrated, adhered and activated on vessel wall. The flow alterations also favor the infiltration of plasma components including LDL, cholesterol and fibrinogen into the arterial wall [40]. Several studies have proved that NO is a potent regulator of hemorheology. NO donor increased the membrane fluidity of erythrocytes, while NOS inhibitor blocked eNOS activity to increase erythrocyte rigidity [24]. NO precursor can reverse the impaired RBC deformability in hypercholesterolemia [12] and inhibit platelet aggregation to improve hemorheology [25]. In this study, we found that Xuezhikang increased the level of NO metabolites, and improved abnormal hemorheology in atherosclerotic rats. We speculated that the improvement of hemorheology may be connected with the increase of NO metabolites. The pathogenesis of atherosclerosis is complicated and multifactorial and Xuezhikang may exert pleiotropic effects, so both the elevated eNOS-NO and improved hemorheology may be beneficial to atherosclerotic disease.

### Conclusions

We conclude that Xuezhikang up-regulated eNOS expression in vascular endothelia and RBCs, increased plasma NOx and improved abnormal hemorheology in high cholesterol diet induced atherosclerotic rats. The elevated eNOS/NO and improved hemorheology may be beneficial to atherosclerotic disease.

## Supporting Information

Figure S1 **Staining of red blood cell and detection of eNOS on erythrocyte membrane.** No white blood cells and platelets were found in the smear slides of washed RBC (panels A and B, Wright-Giemsa Staining). RBC membranes and aorta endothelia were obtained from normal rats using the method in “Materials and methods”. Western blotting using another anti-eNOS antibody (ab5589, Abcam) as the primary antibody confirmed the presence of eNOS on erythrocyte membrane (panel C).
(TIF)
