# Supplementary figures and images for: Xuezhikang, Extract of Red Yeast Rice, Improved Abnormal Hemorheology, Suppressed Caveolin-1 and Increased eNOS Expression in Atherosclerotic Rats
Source: PLoS One. 2013 May 10;8(5):e62731. doi: 10.1371/journal.pone.0062731 (PMC3651163; doi:10.1371/journal.pone.0062731)

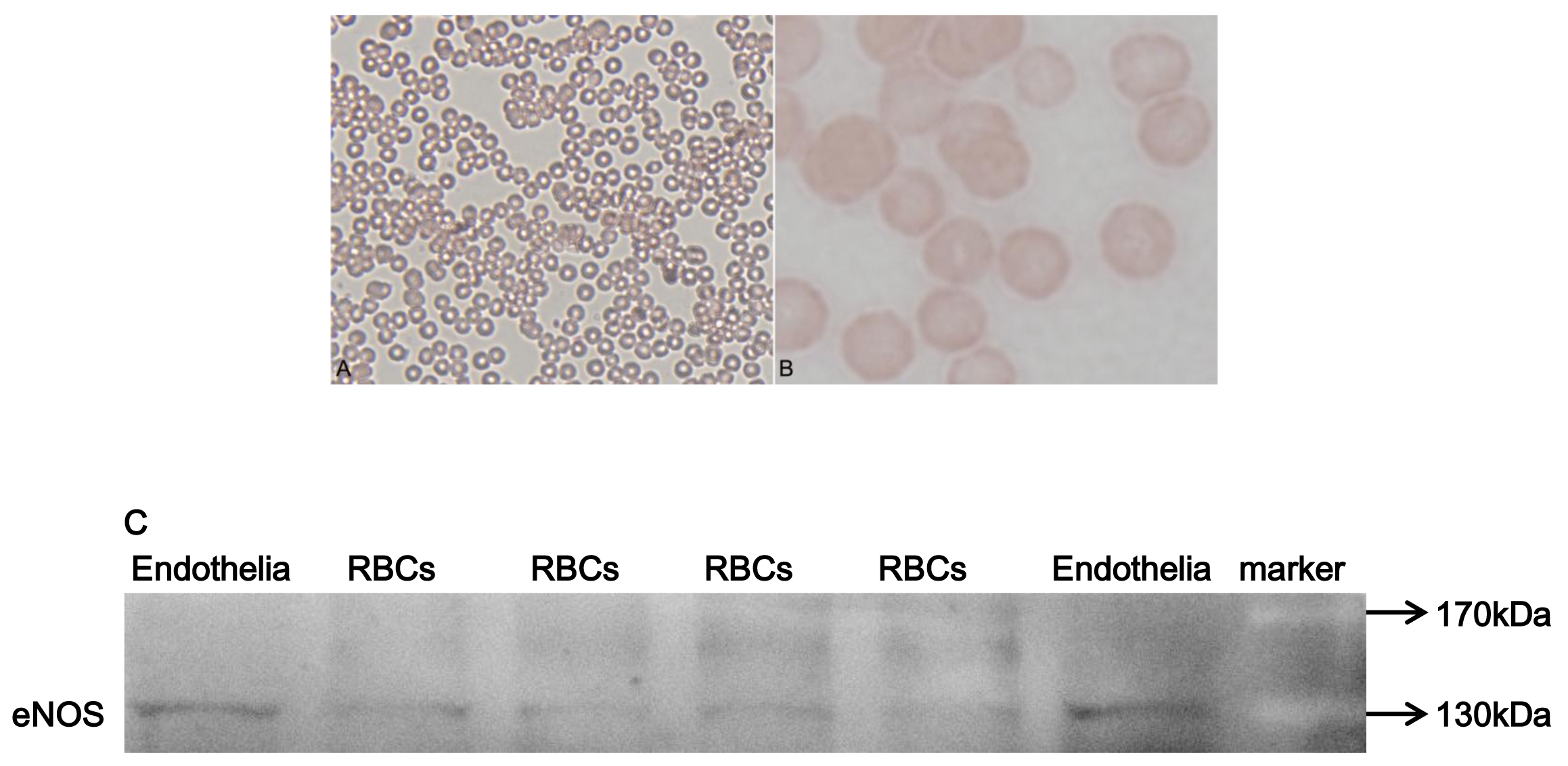

Supplement: Figure S1 — Staining of red blood cell and detection of eNOS on erythrocyte membrane. No white blood cells and platelets were found in the smear slides of washed RBC (panels A and B, Wright-Giemsa Staining). RBC membranes and aorta endothelia were obtained from normal rats using the method in “Materials and methods”. Western blotting using another anti-eNOS antibody (ab5589, Abcam) as the primary antibody confirmed the presence of eNOS on erythrocyte membrane (panel C). (TIF) [file pone.0062731.s001.tif]
